# Uveal Melanoma

**DOI:** 10.3390/cancers11121986

**Published:** 2019-12-10

**Authors:** Ulrich Pfeffer

**Affiliations:** IRCCS Ospedale Policlinico San Martino, 15132 Genova, Italy; ulrich.pfeffer@hsanmartino.it; Tel.: +39-010-555-8462

Uveal melanoma (UM) is among the best characterized solid tumors. It has become clear that there are mainly two classes of uveal melanoma, which can be further subdivided into three or four subtypes that are clearly distinct by their histopathological, cytogenetic, and molecular characteristics. The analysis of the subtypes allows us to prognosticate the metastatic risk of each patient with an unmatched accuracy. While we essentially understand the molecular steps in uveal melanoma carcinogenesis, the mechanisms of metastasization and pronounced liver tropism are still poorly understood [1].

Unfortunately, the diagnostic and prognostic power is not matched by efficacy of therapy. The primary tumor is controlled by radiological and surgical interventions and local relapses are rare. Yet approximately half of the patients develop metastases that rapidly progress to the fatal stage. Despite research, survival of patients with metastatic uveal melanoma has not changed over decades. The identification of the most frequent putative driver mutations, which occur in a mutually exclusive manner in two genes encoding alpha subunits of G proteins, namely G protein subunit alpha Q (GNAQ) and G protein subunit alpha 11 (GNA11) [2,3], has indicated G protein signaling and the activation of MAP kinases as potential targets, but MEK inhibitors have failed to show major effects in clinical trials [4]. More recently, the HIPPO-independent activation of the YAP/TAZ signaling pathway by mutated GNAQ and GNA11 has been described [5,6] but, at present, no specific inhibitors have been tested in the clinics. Recent reports on a specific inhibitor of the mutated form of GNAQ [7,8] must be confirmed and translated into clinical applications. Immune checkpoint blockers that have met considerable success in the treatment of several cancers, including cutaneous melanoma [9], show very low response rates in uveal melanoma (but see [10,11,12,13,14]), likely due to the low number of neo-antigens, a consequence of a very low mutational burden [15,16,17].

Just like for other cancers, the identification of its Achilles’ heel will rely on a deep understanding of the molecular and cellular features of the cancer cell in its permissive microenvironment. This will likely be possible by the thorough molecular characterization of ever more tumors, the development of better cellular and animal models, and the testing of new drugs, whether targeted at the molecular lesions typical of metastatic uveal melanoma or at the immune system.

In the present thematic issue, the authors of 44 articles (31 original research articles [10,13,18,19,20,21,22,23,24,25,26,27,28,29,30,31,32,33,34,35,36,37,38,39,40,41,42,43,44,45,46], 11 reviews [4,11,12,14,47,48,49,50,51,52,53], one position paper [54], and one network report [55]) give insight into the current state of our understanding of uveal melanoma biology and clinics. They also discuss opportunities for the development of new therapeutics that will hopefully soon improve the survival rates of metastatic uveal melanoma patients.

The article collection comprises several reports that address basic biological features of uveal melanoma. Van der Kooij et al. review the differences between cutaneous and uveal melanomas, two tumors that share their origin [51]. Bakhoum and Esmaeli review what the analyses of The Cancer Genome Atlas (TCGA) uveal melanoma data have contributed to our understanding of the biology of this tumor [49]. Pfeffer et al. apply innovative data fusion techniques to the TCGA data in order to combine copy number alteration, DNA methylation and RNA expression datasets for the discovery of subtypes [20].

Piaggio et al. analyze a more extended cohort of 139 cases whose exomes have been sequenced and identify secondary somatic mutations delivering evidence that some of the apparently sporadic mutations that occur in very few or even single cases might contribute to tumor development [18]. Van Poppelen and coworkers analyze somatic mutations in the serine/arginine-rich splicing factor 2 gene (SRSF2) and show a mutational pattern that differs from that observed in myelodysplastic syndrome, where SRSF2 is frequently mutated, likely related to different sets of genes that show aberrant splicing [25].

Weis and coworkers present an epidemiological analysis indicating that the peri-ocular region might have a different or unique exposure pattern to ultraviolet radiation [43].

Pro-tumoral inflammation is addressed by Van Weeghel et al. who show that differences in the inflammatory phenotype and major histocompatibility complex (HLA) expression rely on chromosome 3 status but not on G protein subunit alpha Q (GNAQ) versus G protein subunit alpha 11 (GNA11), mutations in uveal melanoma [27]. Souri and coworkers report that the nuclear factor kappa B (NFkB) pathway is associated with inflammation and HLA Class I expression in UM, and is upregulated when BRCA1 associated protein 1 (BAP1) expression is lost [31]. Souri et al. also show that HLA expression in uveal melanoma is both an indicator of malignancy and a potential target [47]. Wierenga et al. report on tumors in eyes that contain soluble HLA molecules in the aqueous humor that show features of more aggressive tumors and are related to reduced survival [24].

Piquet et al. address the role of hepatic stellate cells in creating a permissive niche for growth and therapy resistance of uveal melanoma metastases [34]. Brouwer et al. report on the association of the hypoxia-inducible factor 1 subunit alpha (HIF1α) and the von Hippel–Lindau protein (VHL) with BAP1 expression, inflammation, and tumor ischemia [36]. Consistent with this observation, Voropaev et al. show that knockdown of the hypoxia mediators cAMP response element-binding protein (CREB) or HIF1α in UM cells by means of replication-competent retroviral vectors dramatically decreases UM tumor progression [32]. Brouwer et al. also address tumor angiogenesis and show that the monosomy 3 and the loss of BAP1 is associated with an increased microvascular density [37]. Van Beek et al. report on rare cases of regional lymphatic spread showing the recruitment of intratumoral lymphatics by uveal melanomas with extraocular extension from subconjunctival lymphatics [45]. Castet et al. review angiogenesis in uveal melanoma and discuss its importance [52].

Dogrusoz et al. show that the DNA-activated protein kinase PRKDC is overexpressed in high-risk uveal melanoma and that the inhibition of such kinases reduces the survival of the tumor cells [30]. Smit et al. identify microRNAs that are associated with uveal melanoma progression through the suppression of stability or translation of mRNAs coding for proteins of various cancer-related pathways [41].

Diagnostic procedures are addressed by Sun et al. who present an innovative artificial intelligence-based method to assess BAP1 expression by immunohistochemistry [19]. Le Guin et al. show that the specific GNAQ Q209R mutation is restricted to circumscribed choroidal hemangioma and very rare in uveal melanoma [46]. Matet and colleagues compare the cytogenetic profiles of choroidal melanoma samples retrieved before and after proton beam irradiation and demonstrate the higher reliability of endoresection material for cytogenetic analysis as compared to fine-needle aspiration biopsy [26]. Anand and coworkers report on a pilot study of circulating tumor cells (CTCs) in early-stage UM that predict an increased risk of metastatic disease [40]. Ferreira et al. provide a dedicated protocol for 3 Tesla magnetic resonance imaging for an improved diagnosis of uveal melanoma [44]. Frizziero et al. review the state of the art of uveal melanoma biopsies [48]. Mariani et al. propose a prognostic nomogram for patients with liver metastases of uveal melanoma to be applied to therapeutic decision-making and risk stratification [39]. Chau et al. propose a guideline for genetic screening of the familial BAP1 tumor predisposition syndrome [29].

Uveal melanoma therapy is addressed by several articles. Fiorentzis et al. propose electrochemotherapy for the treatment of uveal melanoma based on their experience in animal models [21]. Espensen and coworkers explore visual acuity deterioration and radiation-induced toxicity after brachytherapy [28]. Toutee et al. analyze the survival benefit and the risk of visual loss associated with early proton beam radiotherapy [33]. Jochems and colleagues report on treatment strategies and survival of metastatic uveal melanoma patients based on the Dutch Melanoma Treatment Registry [35]. Tura et al. provide data indicating that the therapeutic antibody ranibizumab, and not bevacizumab, suppresses metabolic activity, proliferation, and intracellular Vascular Endothelial Growth Factor A, VEGF-A, levels in uveal melanoma [38]. De Koning et al. report on synergistic effects of poly-ADP ribose polymerase inhibitors and chemotherapy that might rely on inhibition of YAP/TAZ signaling [42].

Immunotherapy, which has shown impressive effects in cutaneous melanoma but much less so in uveal melanoma, is in the focus of several contributions. Rossi and coworkers discuss the immunology of uveal melanoma in order to create a rationale for immunotherapy [11], and Schank and Hassel give an overview of immunotherapies for uveal melanoma [12]. Bol and coworkers present an interesting real-world perspective of therapy with immune checkpoint blockers in metastatic uveal melanoma that indicates some efficacy [13]. Fountain et al. show that immune checkpoint blockers might be useful in the adjuvant setting and call for clinical trials [10]. Damato et al. report on the promising T-cell receptor-gp100 fusion construct tebentafusp as a strategy for adaptive immunotherapy for metastatic uveal melanoma [14].

New targets for therapy are addressed by Rezzola et al. who describe the fibroblast growth factors (FGFs) and their receptors as potential therapy targets in uveal melanoma and show the efficacy of FGF traps [22]. Doherty et al. introduce the DNA-PK as a therapy target since its inhibition leads to increased non-homologous end joining and apoptosis [23]. Vivet-Noguer and colleagues review our knowledge of the molecular biology of uveal melanoma and how this might lead to the identification of new therapies [50]. Violanti et al. offer a different perspective on the molecular oncogenesis of uveal melanoma and the implications for therapy [53]. Croce et al. focus their review on targeted therapies that have not met with success in the clinics and try to give a perspective for future approaches to targeted therapy [4].

Rodrigues et al. provide a position paper of the UM Cure 2020 consortium [54] and Piperno-Neumann et al. report on how the EUropean Rare Adult solid CAncer Network (EURACAN) can be exploited for collaborations on uveal melanoma [55].

This collection of articles yields deep insight into uveal melanoma biology, indicating the routes of further research that will lead to a better understanding of tumor development and relevant, druggable pathways. Therapy of metastatic uveal melanoma remains of very limited efficacy; nonetheless, existing immunotherapy yields some responses. More specific interventions to instruct the immune system will hopefully yield major effects.

The scope of this thematic issue was to bring together experts in the field to sum up their experience and latest findings. Does uveal melanoma generally receive the necessary attention? The analysis of PubMed publications indicates that yes, it does (Figure 1). 

The search terms “uveal melanoma” and “cancer” show similar publication dynamics. Interestingly, in 2015 1,633,390 new cases of cancers were registered in the United States (https://www.cdc.gov/cancer/uscs/about/data-briefs/no3-USCS-highlights-2015-incidence.htm), 3360 of which were uveal melanomas (https://www.cancer.net/cancer-types/eye-cancer/statistics), a ratio of approximately 0.0021 that compares to the ratio of approximately 0.0023 of “uveal melanoma” over “cancer” publications. At present, the clinical trial database (https://clinicaltrials.gov/; interrogated on 18 November 2019) lists 71,324 clinical trials, 148 of which include uveal melanoma patients, a ratio of approximately 0.0021. 

While this is reassuring, the limited progress in uveal melanoma therapy remains alarming and, perhaps, much more attention should be dedicated to this rare but aggressive disease. The present thematic issue will certainly contribute to a better understanding of the peculiarities of uveal melanoma and, hopefully, will also help to make a much-needed step forward in its therapy. 

## Figures and Tables

**Figure 1:**
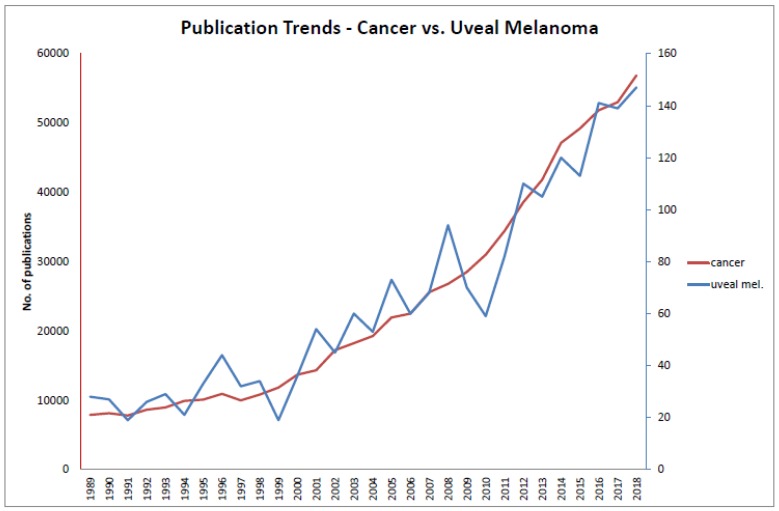
Publication trends. Numbers of publications listed in PubMed in the last 20 years are shown for the search terms “Cancer” (**left** *y*-axis) and “Uveal melanoma” (**right** *y*-axis).
